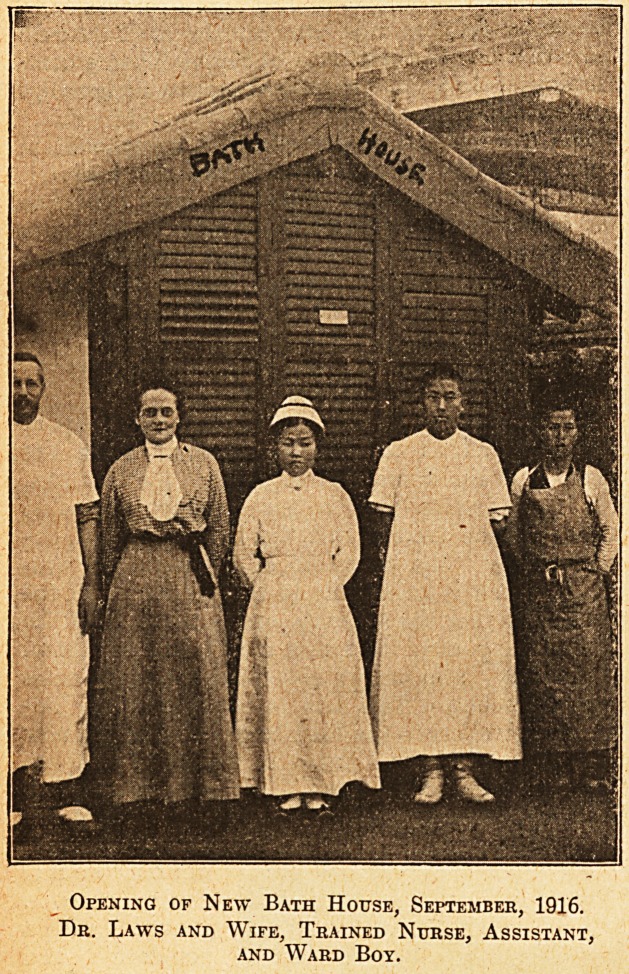# Life in a Korean Hospital

**Published:** 1917-06-23

**Authors:** 


### LIFE IN A KOREAN HOSPITAL.

### A Medical Mission in the East.

If it were not for the medical department of the Society for the Propagation of the Gospel, English people would hear very little of hospital life in the distant places of the world. But, by means of the gifts which are dispatched from England,

OUT-PATIENT BEING "DRESSED."

a tie exists between the Mother-country and remote hospital stations, and the acknowledgments which these gifts produce does something to remind English people of hospital life on the fringe of civilisation. Possibly the medical department alluded to might do more than it does to maintain Englishmen's interest in these curious centres of institutional life, which, after all, represent the least controversial side of mission work. However, from time to time the official silence is lifted by the arrival of a communication to a private donor in this country, and it is the gist of such a welcome communication which THE HOSPITAL prints to-day.

PATIENTS SITTING ON THE VERANDAH OUTSIDE THEIR ROOMS ENJOYING THE SUNSHINE.

The Ay-in Hospital, Chinchun, Korea, is one of the institutions associated with the S.P.G. Mission to Korea, and the limitations imposed by the war are apparent from the gratitude recorded by Dr. Arthur F. Laws on receiving, through the Society's medical department, a gift of three blankets from an English donor. Korea is 12,000 miles away from English blankets, which are at present unobtainable, and Dr. Laws records with humorous aptness that the annals of Yarmouth show that one of the effects of Kubla Khan's over-running of Asia was to raise the price of herrings in the English port!

The mission station consists of a compound, in which the church, the presbytery, the hospital, and a boarding-house or hostel for boys attending the Government primary school are all enclosed. This station is the centre to which twelve stations are affiliated, and in normal times two priests reside there. Dr. Laws first landed in Korea twenty years ago, and his wife only sees another white woman twice a year, when a sister arrives to conduct the Confirmation classes.

The mission is so much a part of the hospital that it is difficult to describe one apart from the other. Its aim is not to "anglicise" the Koreans. Dr. Laws states that it has steadily refused to do so, and he records that the faith is taught unshackled by "the trammels of the parochial system," which is unsuitable to the conditions of the place, and that the converts are impressed by the duty of supporting the native clergy, one of whom, by the way, is in charge of a mission twenty miles from Chinchun. This fact is noteworthy, as unbiassed travellers in the East have noted before now the tendency of the Church in Japan to establish "the Church of England" in that country, a tendency, however regarded, which is no doubt due to the denominational rivalry sure to prevail in a populous and westernised mission field.

The hospital, which must be compared to a cottage hospital in England, is an example of native architecture, except that in common with the mission buildings as a whole it possesses the unique distinction of a tiled roof. A glance at the accompanying illustrations shows that the building is that of a typical Korean gentleman's house. The buildings, in fact, were originally a private residence, but on their purchase for the mission were taken down and rebuilt on Dr. Laws' own plans. The houses are mud huts covered with thatched roofs, which rest upon a wooden framework. The floors of the hospital are of stone, on which the native patients sleep, as anything so barbarous and inconvenient as a bedstead is much disliked by the native mind. In cases of fracture, or disease of

Opening of New Bath House, September, 1916. Dr. Laws and Wife, Trained Nurse, Assistant, and Ward Boy.

the spine, the Western practice has to be resorted to, but the bed is regarded as part of the disagreeable treatment necessitated by a painful disease. Under the floors are flues, which appear to be another Western characteristic. People in England commit a grave error of judgment, and, indeed, display a sad want of taste, when sending out a parcel of red blankets. Colours are only worn by juveniles in Korea, a fact which English people never seem to learn. Uniforms are also disliked, perhaps because they are a foreign innovation, but they seem to be put up with, for a Korean nurse in a white uniform is shown in one of the illustrations. The patients naturally prefer their national dress, and, indeed, chiefly favour the hospital because they are treated there much as they would be if they were guests in the house of any Korean gentleman.

The Korean population is wholly agricultural, and nearly always verminous. Patients have, therefore, to be persuaded to endure a Western bath, which is as much an ordeal for them as it would be for an Englishman in a hot climate to go without one. Habit is equally tyrannous in either form. Clean clothes are, therefore, in large demand. The Korean does not object to these, but the dirty cases have to be placed in blankets, which are then put into a bath of carbolic before being wrung out and dried in the sun. The Korean dress consists of padded quilts made of cotton-wool, which is grown in the neighbourhood, covered with calico, which is dyed blue and red. These garments are difficult to clean, which explains why blankets are used for dirty cases.

One of the most engaging qualities of Koreans, at least to sympathetic people, is their curiosity, and to see Dr. Laws as he is, he must be pictured at his typewriter, surrounded by a circle of Koreans, whose questions are so frequent that he finds it more interesting to listen to them than to attempt to pursue his work of writing home to friends in England. If the medical department of the Society for the Propagation of the Gospel would allow more frequent glimpses of hospital life in its mission stations to English institutional workers, those engaged in managing its stations at the end of the world would feel less aloof than they do, and their English colleagues would be able to take a more personal interest in the work than they are able to do at present.

## Figures and Tables

**Figure f1:**
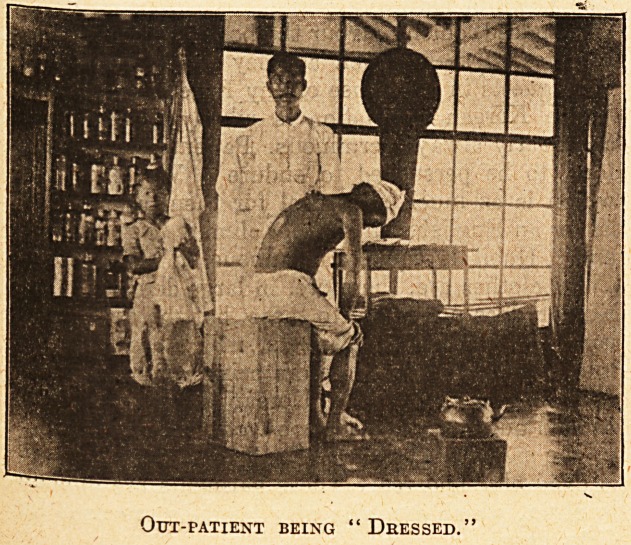


**Figure f2:**
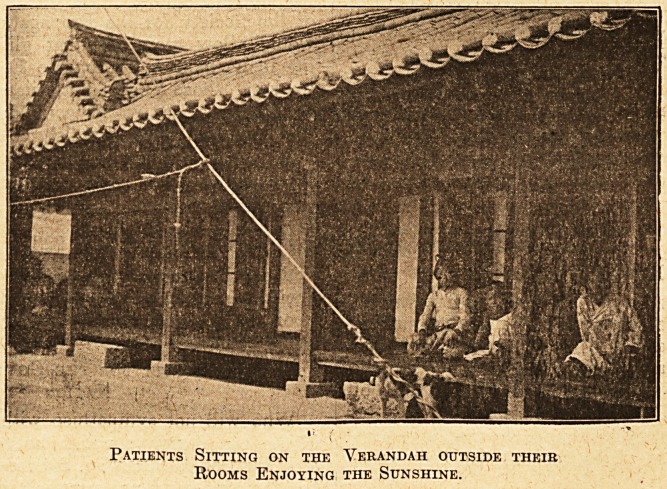


**Figure f3:**